# Analysis of the neurotoxin β-N-methylamino-L-alanine (BMAA) and isomers in surface water by FMOC derivatization liquid chromatography high resolution mass spectrometry

**DOI:** 10.1371/journal.pone.0220698

**Published:** 2019-08-06

**Authors:** Sung Vo Duy, Gabriel Munoz, Quoc Tuc Dinh, Dat Tien Do, Dana F. Simon, Sébastien Sauvé

**Affiliations:** Department of Chemistry, Université de Montréal, Montréal, Quebec, Canada; CEA-Saclay, FRANCE

## Abstract

The neurotoxin β-N-methylamino-L-alanine (BMAA), suspected to trigger neurodegenerative diseases, can be produced during cyanobacterial bloom events and subsequently affect ecosystems and water sources. Some of its isomers including β-amino-N-methylalanine (BAMA), N-(2-aminoethyl) glycine (AEG), and 2,4-diaminobutyric acid (DAB) may show different toxicities than BMAA. Here, we set out to provide a fast and sensitive method for the monitoring of AEG, BAMA, DAB and BMAA in surface waters. A procedure based on aqueous derivatization with 9-fluorenylmethyl chloroformate (FMOC-Cl) was investigated for this purpose. Under optimized conditions, a small aqueous sample aliquot (5 mL) was spiked with BMAA-d3 internal standard, subjected to FMOC-Cl derivatization, centrifuged, and analyzed. The high-throughput instrumental method (10 min per sample) involved on-line pre-concentration and desalting coupled to ultra-high-performance liquid chromatography high-resolution mass spectrometry (UHPLC-HRMS). Chromatographic gradient and mobile phases were adjusted to obtain suitable separation of the 4 isomers. The method limits of detection were in the range of 2–5 ng L^-1^. In-matrix validation parameters including linearity range, accuracy, precision, and matrix effects were assessed. The method was applied to surface water samples (n = 82) collected at a large spatial scale in lakes and rivers in Canada. DAB was found in >70% of samples at variable concentrations (<3–1,900 ng L^-1^), the highest concentrations corresponding to lake samples in cyanobacterial bloom periods. BMAA was only reported (110 ng L^-1^) at one HAB-impacted location. This is one of the first studies to report on the profiles of AEG, BAMA, DAB, and BMAA in background and impacted surface waters.

## Introduction

Cyanobacteria can produce toxic metabolites affecting aquatic and terrestrial wildlife during harmful algal bloom (HAB) events, with potential implications for human health [1,2]. The neurotoxin β-N-methylamino-L-alanine (BMAA) has garnered particular research interests after it was postulated to be a contributing factor in the onset of neurodegenerative diseases [3–6]. BMAA was historically linked to a high incidence of amyotrophic lateral sclerosis–Parkinsonism-dementia complex (ALS-PDC) among the indigenous population of Guam [7–9]. BMAA was produced by the *Nostoc sp*. cyanobacterium, a symbiont within the coralloid roots of the cycad (*Cycas micronesica*). The seeds of this plant were used to produce flour and were also consumed by flying foxes *Pteropus mariannus* that bioconcentrated BMAA and are part of the diet of local residents of the Guam island [7,8].

Various lines of evidence indicate that BMAA is also produced in aquatic environments [10,11]. For instance, BMAA was detected in blooms, scums and mats collected at large spatial scale throughout British water bodies, where cyanobacterial genera such as *Microcystis*, *Nodularia*, and *Planktothrix* were dominant [10]. Virtually all known groups of cyanobacteria from freshwater, brackish and marine environments may in fact be capable of BMAA synthesis, suggesting a potential for ubiquitous distribution [12]. BMAA may also be produced by eukaryotic organisms including Ochrophyta (e.g., diatoms) and Dinoflagellata [7,8].

Among the 260 theoretical structural isomers of BMAA, seven are considered as biologically relevant [13]. In particular, 2,4-diaminobutyric acid (DAB), N-(2-aminoethyl)glycine (AEG), and β-amino-N-methylalanine (BAMA) may be reported in conjunction with BMAA in bloom samples and are increasingly included in environmental monitoring [11,13,14]. The occurrence of DAB is of concern due to its neurotoxicity, potentially leading to the same neurodegenerative diseases as BMAA [15,16]. Limited ecotoxicological data are available for AEG and BAMA, and to the authors’ best knowledge no data is available for other isomers including 3,4-diaminobutyric acid due to the absence of commercial standards [8].

The co-occurrence of isomers of different or no toxicity may be problematic for BMAA analysis and risk assessment, unless highly selective methods are used to allow an unambiguous measurement [17]. Of particular concern is the chromatographic separation, due to the low molecular mass and highly polar nature that precludes a direct analysis by reverse-phase liquid chromatography (RPLC). Alternatively, hydrophilic interaction liquid chromatography (HILIC) was introduced as a powerful method to retain small polar molecules such as BMAA and its isomers [18]. The technique can be hyphenated, for instance, to tandem mass spectrometry (MS/MS) [18,19] and differential mobility MS/MS [20,21]. Capillary electrophoresis coupled to MS/MS was also reported for the quantitative determination of underivatized BMAA [22]. Fast screening techniques such as commercial ELISA kits may not be suitable yet for BMAA analysis [23,24].

Pre-column derivatization techniques can increase analyte hydrophobicity and thus RPLC retention. As reviewed by Cohen [17], BMAA derivatization reagents include, but are not limited to, 6-amino-quinolyl-*N*-hydrosuccinimidyl carbamate (AQC), 9-fluorenylmethyl chloroformate (FMOC-Cl), and propylchloroformate (PCF). The use of dansyl chloride was also recently reported [25]. Analysis of derivatized BMAA and its isomers is often conducted using RPLC coupled to fluorescence detection or tandem mass spectrometry [26–28]. As derivatization is often not specific, optimization of chromatographic separation or monitoring of MS/MS fragmentation reactions may be essential to achieve specificity [27]. As noted in a recent review [29], misidentification due to insufficient resolution from coeluting isomers (or interferences) and inadequate method sensitivity are among the most frequently encountered issues in BMAA analytical methods. Among other noted pitfalls [8,29], the reporting of operating procedures may lack in details and method validation be incompletely documented; this can hinder efficient method applicability and transferability.

Because of these uncertainties, widespread occurrence and exposure to BMAA and its isomers (AEG, BAMA, and DAB) has not been confirmed in surface water sources. Earlier studies have demonstrated the presence of the 4 analytes in certain combinations, both in field-collected algal bloom samples [10,14] and laboratory-grown cyanobacterial cultures [30]. Whether these compounds would occur at detectable levels in surface waters remains, however, an open question. Pollutants in environmental surface waters may occur at concentrations in the low part-per-trillion range (ng L^-1^), which often requires the pre-concentration of a large sample volume (typically, 100–1000 mL) through solid-phase extraction [31]. Because only a small fraction of the extracted sample is eventually injected for analysis, such workflows do not fully capitalize on the invested preparation efforts. On-line pre-concentration directly coupled to RPLC-MS/MS was introduced as an attractive alternative for the trace analysis of environmental waters. The technique was successfully applied to diverse contaminants including microcystins [32,33]. To the authors’ best knowledge, there exist no previous reports on the measurement of BMAA and its isomers by on-line pre-concentration.

Here, we investigated a suitable method for the fast and sensitive determination of AEG, BAMA, DAB, and BMAA in background and HAB-impacted surface waters. Pre-column derivatization with FMOC-Cl was applied to the targeted amino acids, for the first time in surface water matrix. We aimed to submit the complex derivatization samples to direct instrumental analysis, the cleanup being executed through on-line pre-concentration and desalting. The optimization of the reaction and ultra-high-performance liquid chromatography high resolution mass spectrometry (UHPLC-HRMS) yielded satisfactory method sensitivity and robustness. Method validation included the determination of limits of detection and quantification (LOD, LOQ), whole-method accuracy, precision, and matrix effects in surface water samples. The method was applied to a range of surface water samples collected at large spatial scale throughout Canada. To our knowledge, this is one of the first studies to report on the environmental occurrence of AEG, BAMA, DAB, and BMAA in background and HAB-impacted surface waters.

## Materials and methods

### 2.1 Chemicals and standards

β-N-methylamino-L-alanine (L-BMAA) hydrochloride (purity ≥ 97.0%) and L-2-2-diaminobutyric acid (DAB) dihydrochloride (purity ≥ 95.0%) were both obtained from Sigma Aldrich (Oakville, ON, Canada). N-(2-aminoethyl) glycine (AEG) was purchased from Toronto Research Chemicals Inc. (North York, ON, Canada), while β-amino-N-methyl-alanine (BAMA) was obtained from the National Research Council of Canada (Halifax, NS, Canada). The isotope-labelled internal standard L-BMAA hydrochloride-d3 (BMAA-d3) was from Abraxis, Inc. (Warminster, PA, U.S.A.). The derivatization agent 9-fluorenylmethyl chloroformate (FMOC-Cl; purity ≥ 98.0%) was from Fisher Scientific (Alfa AeSar). Ammonium acetate for HPLC (CH_3_COONH_4_; purity ≥ 98%) was acquired from Sigma-Aldrich (St. Louis, MO, U.S.A.). Sodium citrate dibasic sesquihydrate (citrate; purity ≥ 99.0%) and sodium tetraborate decahydrate (borate; purity ≥ 99.5%) were acquired from Sigma-Aldrich (St. Louis, MO, U.S.A.). Acetonitrile (ACN), methanol (MeOH) and water of HPLC grade purity were from Fisher Scientific (Whitby, ON, Canada).

### 2.2 Sample collections

**Fig 1:**
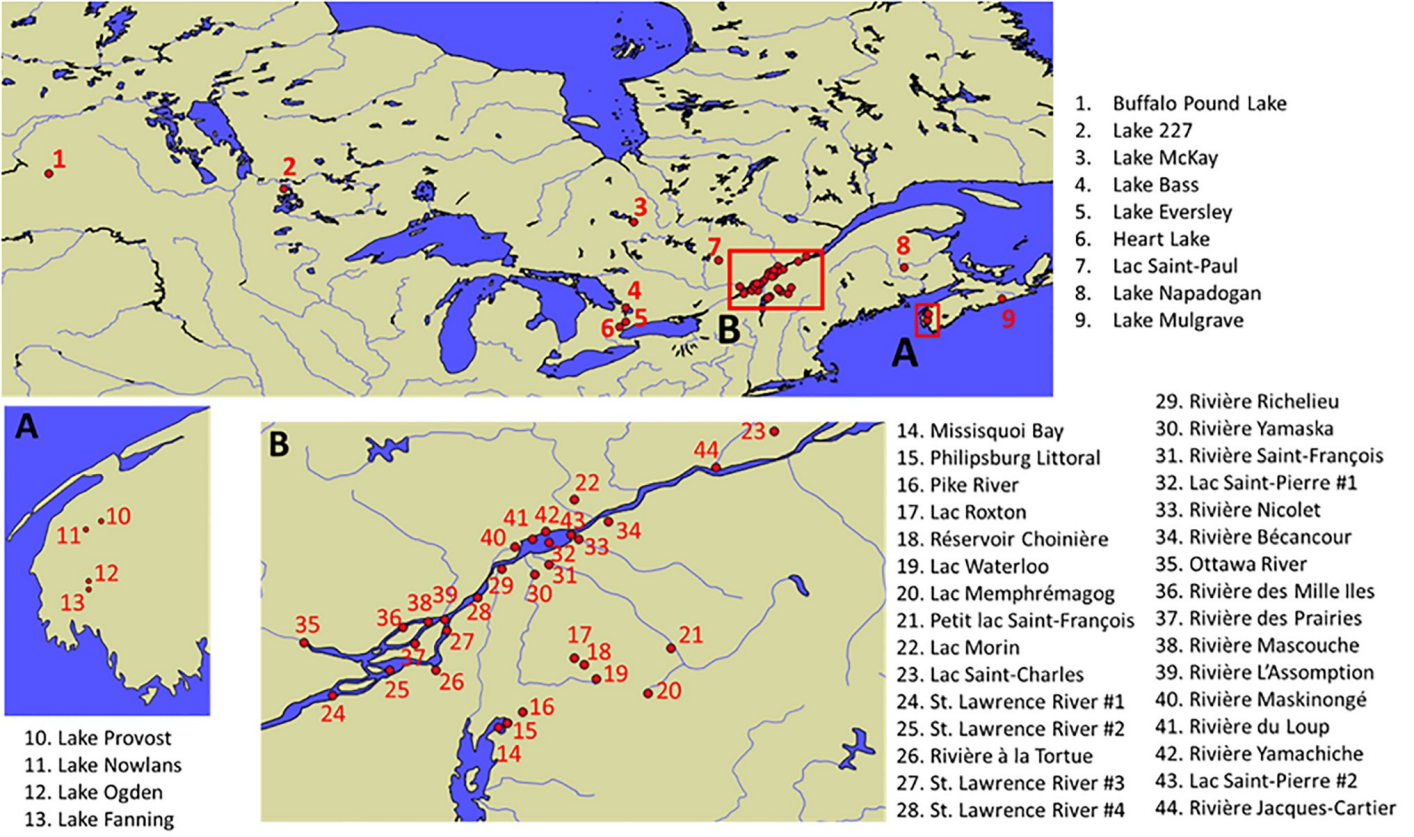
Map of field samples collected in the present survey. Geographical location of the river and lake surface water samples collected at large spatial scale across Canada. Base maps were obtained from Natural Earth (free vector and raster map data available at naturalearthdata.com).

Sample collections of surface water samples (n = 82) were carried out in the 2016–2018 summer seasons in Canada, covering lakes and rivers from the provinces of New Brunswick, Nova Scotia, Ontario, Quebec, and Saskatchewan. Their geographical distribution is illustrated in Fig 1. Most samples were obtained from monitoring under the framework of the ATRAPP project (*Algal Blooms*, *Treatment*, *Risk Assessment*, *Prediction and Prevention through Genomics*) by qualified university personnel and partners. The samples were collected into amber polyethylene terephthalate glycol-modified (PETG) bottles rinsed three times with the site surface water, filled to the brim, and sealed. The samples were stored at -20 °C and shipped to the laboratory within 3 days, where they were submitted to three freeze-thawing cycles and filtered through Acrodisc GH Polypro (GHP) filters (0.2 μm). Some samples from recreational lakes were obtained through a crowd-sourcing initiative (*Adopt a Lake* campaign). The participants received a sampling box with detailed protocols, a sample information sheet to record the necessary data, and the sampling equipment. Additional background surface water samples were obtained from a large scale survey of the St. Lawrence watershed, conducted in the late Summer 2018. Samples from the St. Lawrence River were collected with the *Lampsilis* research vessel (Université du Québec à Trois-Rivières), while other tributaries were sampled near the river mouths [34]. Further details on surface water samples, including locations and corresponding dates of collection, are provided in the Supplementary Information (S1 and S2 Tables). No specific permissions were required for accessing/conducting the field sampling of surface waters, and the field studies did not involve endangered or protected species.

### 2.3 Sample preparation

**Fig 2:**
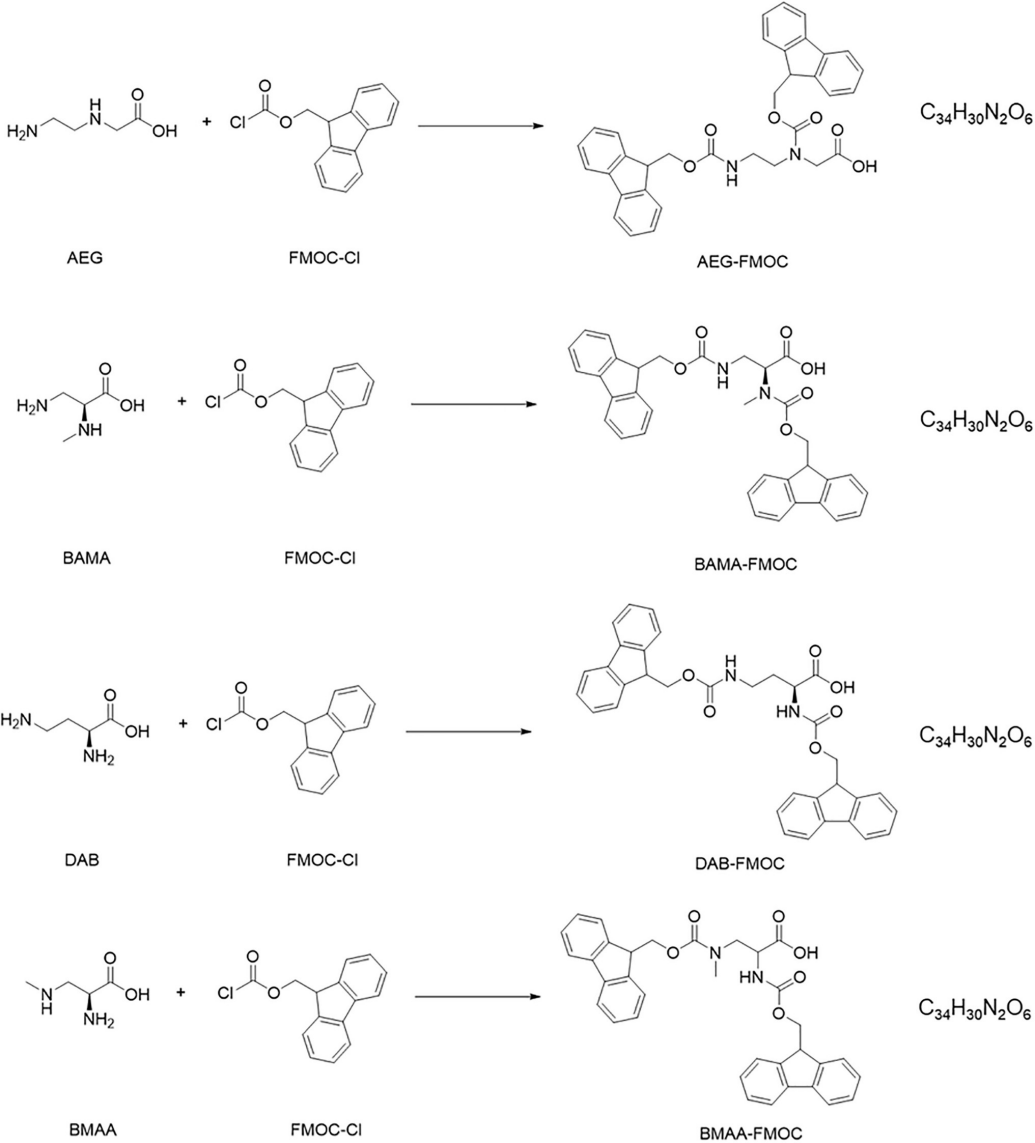
Derivatization reactions of the targeted amino acids with 9-fluorenylmethyl chloroformate (FMOC-Cl).

The preparation of surface water samples was conducted as follows (S1 Fig). A 5-mL aliquot of GHP-filtered surface water was introduced in a 10-mL amber glass vial and spiked with the BMAA-d3 internal standard for a concentration of 200 ng L^-1^ (20 μL of a 50 ng mL^-1^ internal standard solution prepared in HPLC water). The borate (300 μL of a 100 mM solution prepared in HPLC water) and citrate (300 μL of a 150 mM solution prepared in HPLC water) buffers were then added. The mixture was briefly vortexed (10s; 3200 rpm) and a wait time of 5 min was applied. Following the addition of 300 μL of FMOC-Cl (3 mg mL^-1^ in ACN), the sample was briefly vortexed (10s; 3200 rpm) and stirred at 200 rpm (1h, 65 °C, no light) to allow the derivatization reaction to take place (Fig 2). After this step, the samples were left to cool down to ambient temperature and 300 μL of MeOH were added to stop the reaction and minimize the sorption losses of FMOC-derivatized amino acids (Section 3.2). The resulting mixture was briefly vortexed (10s; 3200 rpm) and centrifuged (10 min; 6000 rpm) prior the aliquoting of 5 mL of supernatant into a LC-MS amber glass vial for analysis.

### 2.4 Instrumental analysis

The derivatized samples were processed by on-line pre-concentration and desalting coupled to ultra-high-performance liquid chromatography high resolution Orbitrap mass spectrometry (UHPLC-HRMS), using the EQuan interface and the Chromeleon 7.2 Software (Thermo Fisher Scientific, Waltham, MA, U.S.A., and Dionex Softron GMbH, part of Thermo Fisher Scientific, Germany). A PAL RTC autosampler (Zwingen, Switzerland) was used for sample injection into a 2-mL stainless steel (SST) loop. A Thermo Dionex UltiMate^™^ 3000 pump was used for on-line pre-concentration and desalting, and a Thermo Dionex UltiMate^™^ 3000 RS pump for subsequent UHPLC separation.

The pre-concentration step consisted in loading 2 mL of the sample onto a Thermo HyperSep Retain PEP column (hydrophilic lipophilic balance, 20 mm x 2.1 mm, 40–60 μm particle size) at a flow rate of 2,000 μL min^−1^. After the loading step, the on-line aqueous mobile phase (HPLC water) was left to flow for 1 min at 2,000 μL min^−1^ to allow salt removal. Analytes were then back-flush eluted at 450 μL min^−1^ with the analytical mobile phase (A: 2.5 mM CH_3_COONH_4_ in HPLC water; B: ACN). Chromatographic separation was achieved by a Thermo Hypersil Gold C18 column (100 mm x 2.1 mm, 1.9 μm particle size, 175 Å pore size) thermostated at 35 °C and fitted with a 0.2 μm column pre-filter. Details on the chromatographic gradient elution programs are provided in SI (S3 Table).

The FMOC-derivatized analytes were detected in negative electrospray ionization mode using a Thermo Q-Exactive Orbitrap mass spectrometer (Thermo Scientific, San Jose, CA, U.S.A.) operated in full scan MS mode (scan range: 200–600 m/z). Orbitrap parameters were as follows: resolution was set at 70,000 full width at half maximum (FWHM; value at 200 m/z), automated gain control (AGC) at 3E6, and injection time (IT) at 100 ms. Further details including source parameters are provided in SI (S3 Table). LC-MS data quantification was carried out using Xcalibur 3.0 software (Thermo Scientific).

### 2.5 Method validation

The method was validated following the literature and general guidance documents [29,35–37]. Validation parameters including linearity, method limits of detection (LOD) and limits of quantification (LOQ), accuracy, precision, and matrix effects were assessed. These tests were performed by spiking surface water with native analytes and the internal standard at the beginning of the sample preparation procedure. They therefore represent an evaluation of the method as a whole (sample preparation + instrumental analysis) and using real matrix samples. A description of the quantification procedure is further provided in Section 3.5.

Calibration curves were constructed in a composite surface water matrix (using pooled samples from the present survey) by adding the native analytes at 7 levels between the LOQ and 1,000 ng L^-1^ (internal standard set at 200 ng L^-1^). Compound-specific linearity range and determination coefficients (R^2^) were recorded, as were deviations (bias %) between expected and calculated-back concentrations (Section 3.6).

Method LODs were evaluated as 3 times the standard deviation of 10 blank surface water samples spiked at low level (10 ng L^-1^) with target analytes (EURACHEM method [38]). The method LOQ was determined from a similar process but applying a different multiplier (k_Q_) to the standard deviation (EURACHEM method [38]). The k_Q_ multiplier was selected (fit-for-purpose approach) so that the conditions for acceptable accuracy (70–120%) and precision (RSD <20%) performance were satisfied at the set LOQ [39,40].

Filtration pre-treatment of surface water samples was performed and recoveries were assessed for the following syringe filter types: Millipore nitrocellulose (NC, 25 mm diameter, 0.22 μm porosity), Kinesis KX glass fiber (GFF, 13 mm diameter, 0.22 μm porosity), and Acrodisc GH polypro (GHP, 25 mm diameter, 0.2 μm porosity). For each filter type, we used a background surface water sample (free from suspended particles) spiked at 50 ng L^-1^ with native analytes and passed through the syringe filter (n = 3 per filter). The BMAA-d3 was added to these samples post filtration. Three nonfiltered surface water samples spiked with both native analytes and BMAA-d3 were used as a reference to determine the recovery. The different sample types were submitted to the derivatization and LC-MS analysis, and filtration recovery (%) was determined based on the analyte to internal standard area ratio of the filtered sample, divided by the average area ratio of the nonfiltered reference.

Whole-method accuracy was assessed at two spike levels (QC_1_: 75 ng L^-1^ and QC_2_: 750 ng L^-1^) within the linearity range but not previously included in the calibration curve regression. The accuracy was determined as per Eq (E1) as follows [40]:

$$Accuracy\;(\%)=(C_{measured}/C_{expected})*100$$ (E1)

Where C_measured_ represents the concentration quantified using the matrix-matched calibration curve, and C_expected_ represents the theoretical concentration spiked to the sample.

Intraday and interday precisions were assessed at two spike levels (QC_1_: 75 ng L^-1^ and QC_2_: 750 ng L^-1^). The intraday precision was derived from the relative standard deviation (RSD, %) of replicate (n = 5) samples prepared and analyzed within a single work day. This process was repeated on a second (n = 5) and third (n = 5) work days, and interday precision derived from the overall RSD (n = 15).

It should be mentioned that the absolute derivatization yield could not be determined, due to the lack of a FMOC-BMAA standard. However, the calibration curve (with natives and BMAA-d3 internal standard) was submitted to the whole process, implying that any bias resulting from the derivatization yield was corrected by the quantification procedure. Sample-to-sample variations of derivatization yields can also be controlled upon verification of the absolute area of FMOC-derivatized BMAA-d3 across samples.

Sample-to-sample matrix effects [41] were assessed for a subset of surface water samples, by performing standard additions to the particular sample, compared to the calibration curve used for quantification, as per Eq (E2) as follows [42]:

$${Matrix\;effect}_{sample\;i}(\%)=({SA}_{sample\;i}/REF-1)*100$$ (E2)

Where SA_sample i_ represents the slope of the standard additions to the particular surface water sample, and REF represents the slope of the calibration curve routinely used for quantification (composite matrix-matched curve). In all cases, the native analytes and BMAA-d3 internal standard were added to the test samples at the start of the preparation procedure, and therefore represent whole-method matrix effects.

### 2.6 Quality assurance and quality control

The identification of FMOC-derivatized analytes in field samples relied on matching retention times with certified standards and exact mass accuracy of extracted full scan MS chromatograms within a ±5 ppm tolerance window [43].

Injection blanks (HPLC water aliquots directly submitted to instrumental analysis) were injected at the start of each LC-MS batch sequence and after the high-end calibration curve level to control the absence of carryover. Method blanks were executed for each preparation batch of samples, consisting in HPLC water spiked with BMAA-d3 internal standard at 200 ng L^-1^ and submitted to derivatization and instrumental analysis as described in Sections 2.3 and 2.4. None of the targeted analytes were detected in injection blanks and method blanks.

A matrix-matched calibration curve (surface water) was executed at the start of each LC-MS sequence (initial calibration) and was required to meet acceptance criteria [44]. A duplicate set of quality control samples was also run and quantified against the calibration curve to verify the accuracy (initial calibration verification–ICV standards). In addition, duplicate continued calibration verification (CCV) standards were run after every 15 samples to control the accuracy along the analytical sequence. Both standard types, ICV and CCV, were constructed in surface water at an intermediate concentration level (100 ng L^-1^) for the targeted analytes, while the internal standard was set at 200 ng L^-1^. The accuracy of ICV and CCV standards was required to fall within the acceptable range of 70–120% [35].

The recovery of FMOC-derivatized BMAA-d3 internal standard was also monitored for CCV standards and field samples [40]. When the calculated concentration in field samples was above the upper calibration curve level, or if a drift in internal standard recovery was noted, a new sample preparation was performed applying a dilution factor (typically, 2x) before the FMOC derivatization process.

## Results and discussion

### 3.1 Optimization of sample preparation

Previously, Kisby et al. [45] introduced a method based on precolumn FMOC derivatization and liquid chromatography with fluorescence detection as a means to analyze BMAA in mammal tissues and *Cycas circinali*s seeds. Based on analytical methods [46,47] to determine glyphosate (an aminophosphonic analog of the naturally-occurring amino acid glycine), we surmised that the FMOC-derivatized BMAA (Fig 2) could be amenable to LC-MS detection. Pre-column FMOC derivatization has not been investigated for ultra-trace LC-MS analysis of BMAA in environmental surface waters, and thus requires appropriate optimization.

A summary of the experimental design applied for optimization of sample preparation is provided in Table 1. Common to all tests, we used replicates (n = 3 per condition) of background surface water samples from the St. Lawrence River spiked at 1,000 ng L^-1^ with BMAA. These samples were submitted to the derivatization procedure prior analysis by on-line pre-concentration coupled to UHPLC-HRMS. At the initial stage of the optimization process, we selected FMOC and buffer concentrations from a published method for trace analysis of glyphosate in surface water, while the reaction time was set at 24h [34]. Because of the low hydrosolubility of the derivatization agent, some previous studies conducted FMOC derivatization of amino acid-related compounds using ratios of acetonitrile to water as high as 50:50 (v/v) [48]. However, lower ratios have also been employed with little to no effect noted [49]. Considering the need for a highly aqueous sample to perform large volume on-line pre-concentration during instrumental analysis, we selected an initial acetonitrile to water ratio of 10:90 (v/v). Preliminary experiments were first performed to explore the effects of variable reaction times (3h *vs*. 24h) and reaction temperatures (40–70 °C), resulting in higher FMOC-BMAA signals when a shorter reaction time was combined with a 60 °C temperature (unpublished data).

**Table 1:** Optimization of sample preparation. Experimental design applied in the optimization of sample preparation conditions. Bold font indicates the parameter(s) being tested for each experiment.

	Experiment number						
	1	2	3	4	5	6	Selected
% ACN for reaction	10%	**5–15%**	5%	5%	5%	5%	5%
Reaction temperature	**40–70 °C**	60 °C	**60–70 °C**	65 °C	65 °C	65 °C	65 °C
Reaction time	**3h or 24h**	3h	**0.5–3 h**	1h	1h	1h	1h
Reaction stirring speed	200 rpm	200 rpm	200 rpm	200 rpm	200 rpm	200 rpm	200 rpm
Borate concentration	150 mM	150 mM	150 mM	**50–150 mM**	100 mM	100 mM	100 mM
Citrate concentration	150 mM	150 mM	150 mM	150 mM	**100–250 mM**	150 mM	150 mM
FMOC-Cl concentration	30 mg/mL	30 mg/mL	30 mg/mL	30 mg/mL	30 mg/mL	**1.5–30 mg/mL**	3 mg/mL

The ACN percentage used for the reaction was investigated at three discrete levels (Experiment #2). Note that the final ACN percentage prior LC-MS analysis was adjusted to 15% in all samples, so that differences between conditions would arise from the actual derivatization reaction and not from variable retention efficiency during on-line solid phase extraction. Varying ACN in the 5–15% range prior reaction had no significant effect on the FMOC-BMAA absolute area (S2 Fig). The variability was typically lower with 5% ACN (RSD = 7%) compared to the other two tested levels (RSD = 25–39%), which led to the choice of the former condition.

The use of MeOH for organic solvent amendment prior the reaction is generally avoided due to potentially enhanced solvolysis of FMOC-Cl compared to other solvents such as ACN or acetone [50]. MeOH could still prove useful in quenching the medium post derivatization. Post-reaction conditions were therefore investigated, including different storage temperatures and MeOH percentages. Our results indicate that these parameters are not major influential variables of FMOC-BMAA signal over the tested ranges (S3 Fig).

**Fig 3:**
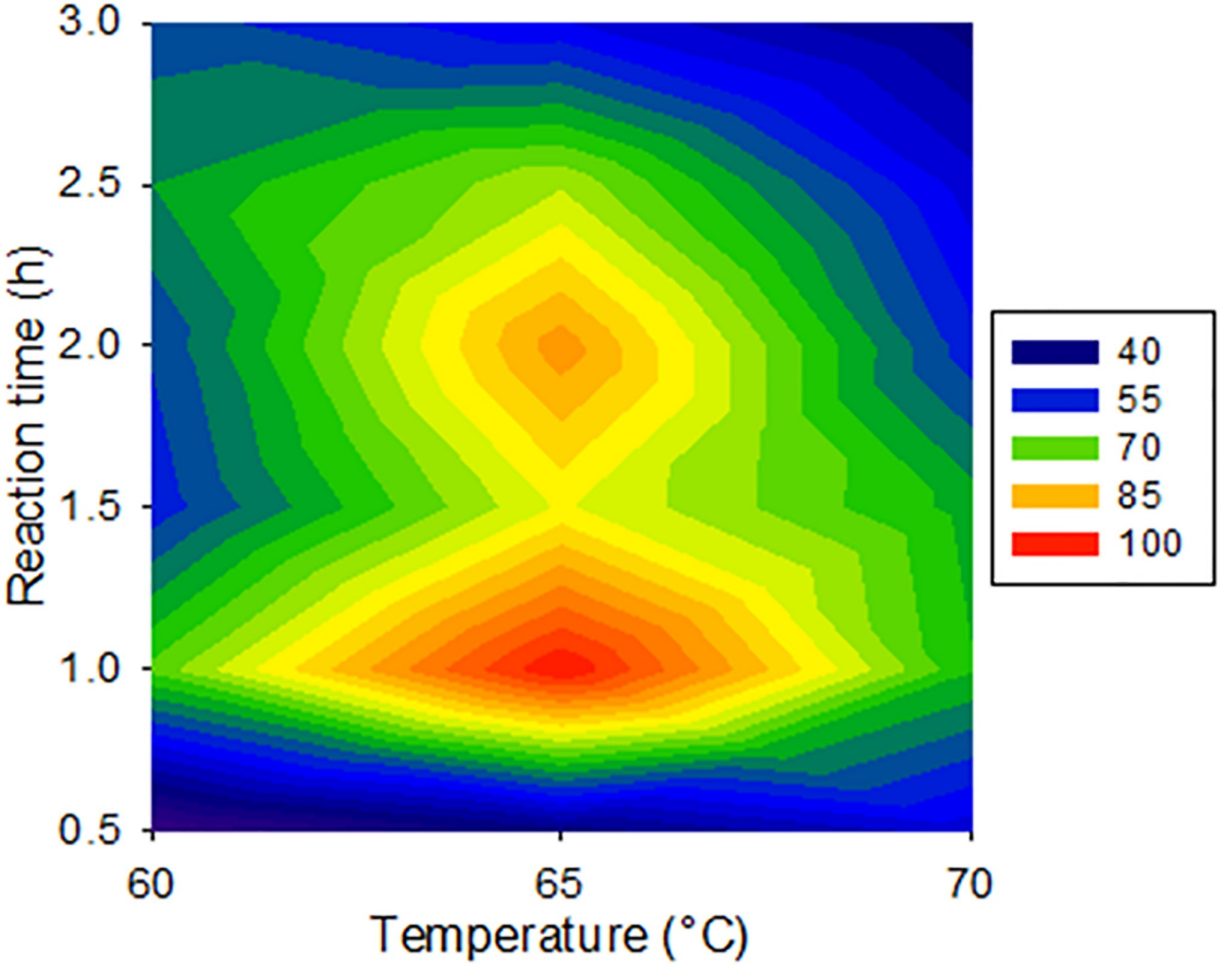
Optimization of derivatization reaction time and temperature. Response surface of BMAA signal upon full-factorial experimental design optimization of FMOC derivatization reaction time and temperature. FMOC-BMAA areas were normalized to the observed maximum (maximum set to 100%) across the 18 tested combinations (n = 3 per condition).

In view of the above, we selected an initial ACN to water ratio of 5:95 (v/v) during the reaction, applied a 15-min wait time at ambient temperature to let the samples cool down after derivatization, and further introduced 5% MeOH (v/v) to quench the reaction. Other derivatization conditions were also investigated. The stirring speed applied during the reaction was varied between 150 and 200 rpm; the latter condition was selected for the lower variability it yielded. Derivatization reaction time and temperature were jointly investigated using a full-factorial experimental design (Experiment #3). A response surface modelling was applied to the analyte response after normalization to the maximum observed condition [51]. Regardless of temperature, higher analyte response was typically obtained with a 1-h reaction time (Fig 3). This agrees with FMOC derivatization times of amino acid analogs in natural surface waters [46,52].

Using the retained reaction conditions (1h; 65°C), the concentrations of citrate and borate buffers were varied (Experiments #4–5). Some FMOC derivatization procedures favor the use of ethylene diamine tetraacetic acid (EDTA) as chelating agent to allow the complexation of metallic cations [34] that could otherwise affect the reaction yield [53]. In the present study, however, EDTA was shown to induce a slight yet systematic contamination of matrix blanks with AEG. Based on earlier literature [54,55], this artifact may be attributed to the decomposition of EDTA under the heating conditions (S4 Fig), and we alternatively used citrate buffer to allow metal complexation. We selected a citrate buffer concentration of 150 mM, which provided a higher FMOC-BMAA signal and acceptable variability (S5 Fig). The borate buffer was used to provide basic conditions for the FMOC derivatization reaction to take place; a 100 mM concentration was finally selected (S6 Fig). The FMOC-Cl concentration was varied at 8 discrete levels between 1.5 and 30 mg mL^-1^ (Experiment #6). Lowering the FMOC-Cl concentration from 30 to 3 mg mL^-1^ translated into a 6-fold increase in analyte signal (S7 Fig), presumably due to lower instrumental matrix effects. The addition of a wash step consecutive to sample loading also contributed to salt and matrix removal (Section 3.3).

### 3.2 Selection of suitable chromatographic conditions

The most challenging step in the analysis of BMAA is, by far, its resolution from AEG, BAMA, and DAB isomers [13]. We first investigated a suitable column for the separation of FMOC derivatives. A preliminary test was conducted with 5 mM of CH_3_COONH_4_ in the aqueous mobile phase and using a C18 column (2.1 mm x 50 mm, 1.9 μm particle size), but the compounds were not chromatographically resolved. The use of a longer C18 column (2.1 mm x 100 mm, 1.9 μm particle size) improved the separation, while a pentafluorophenyl (PFP) column of similar characteristics (2.1 mm x 100 mm, 1.9 μm particle size) did not. To improve the separation with the 100-mm C18 column, the chromatographic gradient was slowed until all compounds had eluted, and the mobile phase flow rate and column temperature were also reduced (S3 Table). The selected conditions produced acceptable separation between the 4 isomers, which eluted as follows: AEG, BAMA, DAB, and BMAA.

Improvement in analyte separation and response was further tested with mobile phase variations. Isopropanol was amended (10% v/v) to the ACN organic mobile phase to lower the eluting strength and, as anticipated, the retention time of targeted analytes increased (unpublished data). However, this also translated in higher pressure (due to the higher viscosity) and less resolved peaks, and the use of isopropanol was therefore avoided. We also tested mobile phases without ammonium acetate (i.e., A: HPLC water, B: ACN) which resulted in poor retention and separation, indicating that CH_3_COONH_4_ may be essential to achieve the intended purpose. Ammonium acetate was previously reported to improve the chromatographic separation of difficult-to-analyze compounds, including human metabolites (e.g., uroporphyrins) and flavonolignans (e.g., isosilybin A and B) of *astaraceae* plant extracts [56,57].

**Fig 4:**
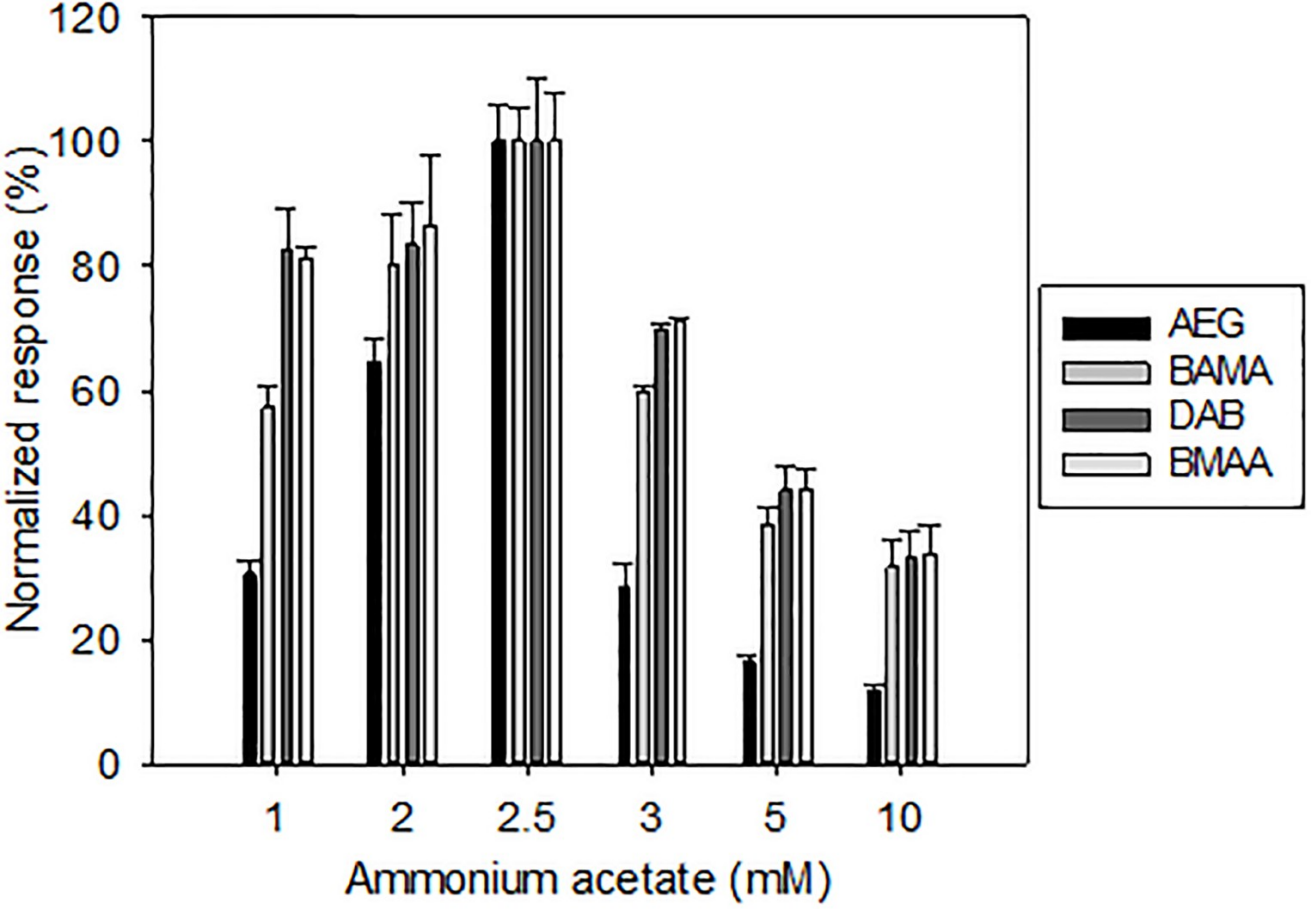
Influence of UHPLC mobile phase modifier. Influence of the concentration of ammonium acetate in the UHPLC mobile phase on signals of FMOC-derivatized AEG, BAMA, DAB, and BMAA. For each compound, the absolute area was normalized to the observed maximum (maximum set to 100%) across the tested conditions. Error bars represent standard deviations (n = 3).

**Fig 5:**
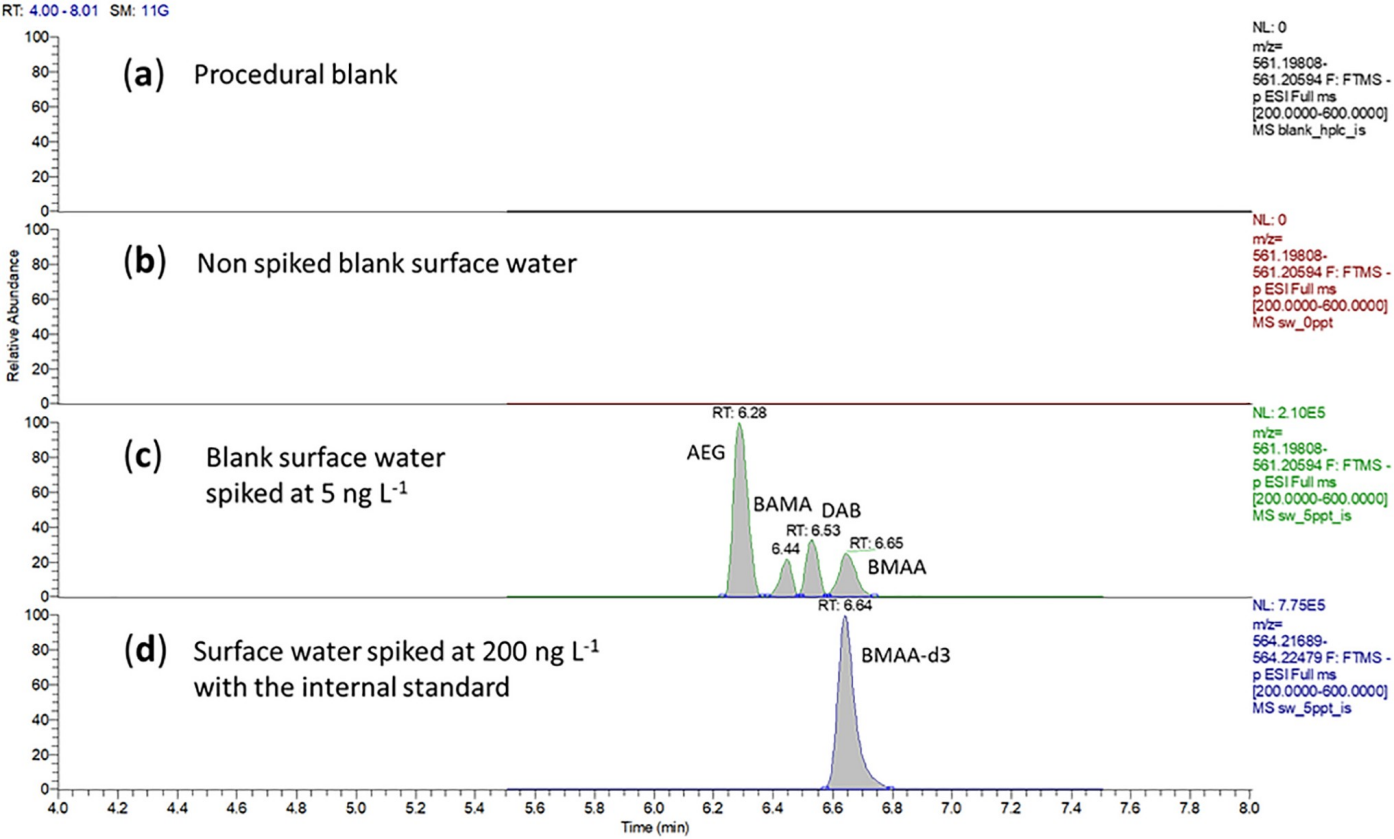
Chromatographic performance of the optimized method. Extracted chromatograms obtained using the present FMOC derivatization–on-line pre-concentration and desalting–UHPLC-HRMS method, including derivatized BMAA and isomers in (**a**) method procedural blank, (**b**) blank surface water matrix, (**c**) blank surface water matrix spiked at 5 ng L^-1^ with the targeted analytes, and (**d**) blank surface water spiked at 200 ng L^-1^ with the internal standard.

Acceptable separation of the 4 isomers was observed when CH_3_COONH_4_ was set at 2 mM or higher (Fig 4). Although we observed higher retention and separation with increasing ammonium acetate concentrations, we finally selected a 2.5 mM concentration due to the substantial signal decrease at higher concentration levels (Fig 4), presumably reflecting decreased ionization efficiency. Using the optimized conditions, suitable sensitivity and chromatographic separation were achieved (Fig 5).

### 3.3 Optimization of on-line pre-concentration and desalting

The other challenging step specific to the present study lies in the direct injection of large sample volumes with a high salt content. Liquid-liquid extraction or off-line solid-phase extraction can be used to obtain cleaner extracts. However, such strategies also require longer sample preparation times and the recovery may be adversely affected during the multi-step process. On-line pre-concentration of underivatized surface water samples was successfully achieved in recent years to analyze microcystins [32,33], and we demonstrate here the suitability of the approach to FMOC-derivatized BMAA and its isomers.

A fully automated clean-up method coupled to LC-MS appears as an attractive alternative, but there are some caveats. On-line pre-concentration is performed using a chromatographic column with a valve-switching process for elution and transfer of the retained analytes onto the analytical column. The on-line column sorbent nature should, therefore, be selected to ensure sufficient retention of the targeted analytes at the sample loading step, all the while allowing their subsequent elution with the mobile phase gradient. Counterintuitively, a strongly hydrophobic sorbent is therefore not always the best option for hydrophobic analytes such as FMOC derivatives. In our case, a hydrophilic-lipophilic balance sorbent (HyperSep Retain PEP) provided indeed higher signals (~1.8x) than a hydrophobic (C18) sorbent, likely due to the excessive adsorption of analytes on C18 (S8 Fig).

Following pre-concentration, the aqueous on-line mobile phase (H_2_O) may be left to flow through the on-line sorbent to allow matrix and salt removal [58]. This washing step, intercalated between the pre-concentration and elution steps, is the driving factor of the on-line desalting process. The wash volume should be optimized to obtain efficient desalting and avoid breakthrough losses of targeted analytes [59]. We observed an increase in analyte signal from 0.5 to 2 mL wash volume with marginal variations afterwards, which led to the selection of a 2-mL wash volume (S9 Fig). The loading (and wash) speed is another critical parameter of the on-line process. Ideally, flow rate should be high enough to expedite pre-concentration (and desalting) and thus allow a faster method run time. Over the range of investigated values (from 1,000 to 2,500 μL min^-1^), we selected a 2,000 μL min^-1^ flow rate that yielded the best compromise in terms of analyte signal, variability, and sample throughput (S10 Fig).

### 3.4 Assessment of filtration and other sorption artifacts

**Fig 6:**
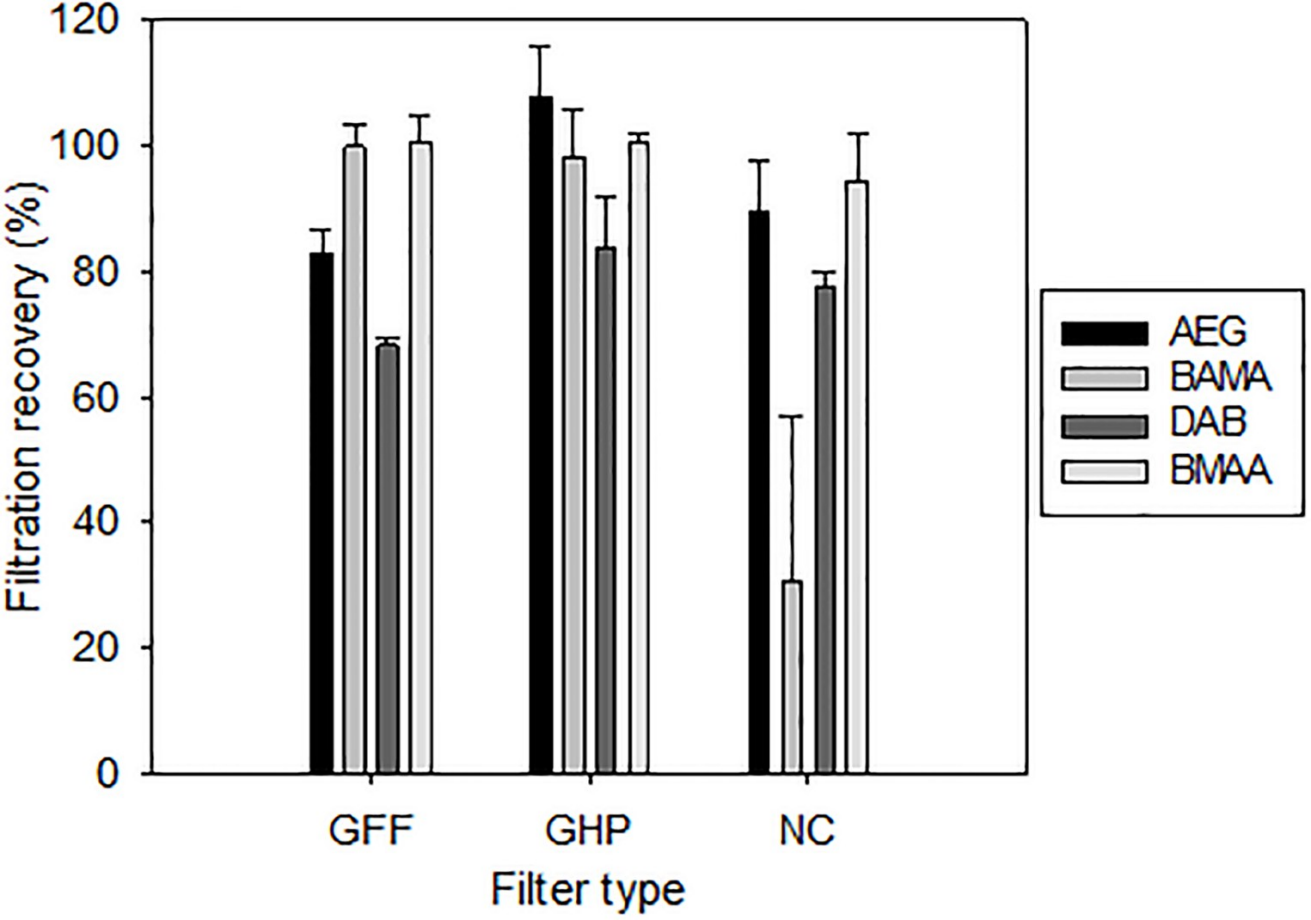
Assessment of filtration recovery. Influence of filter type on analyte recovery (%) during the surface water pre-filtration step. The detailed procedure is highlighted in Section 2.5. Error bars represent standard deviations (n = 3).

Filtration may be used as an initial pre-treatment step of surface water samples. Whether this step could cause unintended analyte losses should, however, be studied (Fig 6). In view of the polar nature of underivatized amino acids, three filtration materials were tested, including nitrocellulose (NC), glass fiber (GF/F) and GH Polypro (GHP, hydrophilic polypropylene). Regardless of filter type, we observed satisfactory filtration recoveries of BMAA (94±8%, 101±4% and 100±1% for nitrocellulose, GF/F, and GHP, respectively). This agrees well with previous studies which employed nitrocellulose or GF/F materials as a pre-treatment filtration step of environmental waters [60,61]. The GHP filter was selected for the consistently high recoveries it yielded for AEG, BAMA, DAB, and BMAA (range: 84–108%), but the use of glass fiber would remain an equally valid alternative (Fig 6).

Filtration could also be seen as a simple way to remove precipitates following the derivatization reaction and ensure a robust analysis during long LC-MS batch sequences. Inconsistent filtration recoveries were, however, noted when passing the derivatized samples through diverse filter types (unpublished data). We ascribe this to the possible sorption of derivatized BMAA and its isomers onto the plastic surfaces of filtration materials (e.g., syringe, filter capsule) due to the hydrophobic nature of the compounds grafted with two FMOC moieties. In view of this, the filtration of derivatized samples was avoided and we used instead high-speed centrifugation prior to aliquoting and analysis.

The use of plastic materials was avoided as much as possible in the procedure to minimize sorption losses of the FMOC-derivatized analytes. For instance, the Peek sample injection loop was changed for a 2-mL stainless steel (SST) loop, resulting in an improvement in sensitivity between 1.66× and 1.79× (S11 Fig). Using the optimized method, we did not observe any time-dependent artifacts when injecting samples at different wait times following aliquoting for LC-MS analysis (S12 Fig), suggesting suitability for analyses of large sample series.

### 3.5 Quantification procedure and matrix effects

A common preconception is that LC-MS techniques guarantee selectivity and accurate measurements of analyte concentrations. However, this premise may be challenged by ionization competition phenomena, particularly when analyzing complex samples [62]. There are several schools of thought regarding the quantification strategy, implying different calibration curve types. In the present study, in view of the complexity of derivatized samples directly submitted to instrumental analysis, a matrix-matched calibration curve was used with further internal standard correction.

We proceeded as follows for the quantification. Surface water samples were pooled to create a composite matrix-matched sample. Calibration curve levels were created by adding incremental levels of native analytes to 5 mL of the composite sample (while the internal standard was set at 200 ng L^-1^). The calibration levels were then submitted to the same FMOC derivatization process and analysis as the field samples. The concentration in field samples was derived from the native analyte to BMAA-d3 area ratio noted in the particular field sample, divided by the slope of the matrix-matched internal calibration curve (inverse-weighted linear regression of A_native_/A_BMAA-d3_ = f(C_native_)) [42].

Besides, we assessed the *relative matrix effects* by performing standard additions on individual surface water samples from different locations, and comparing the slope to that of the composite matrix-matched curve (Section 2.5). This test was designed to ascertain whether matrix effects could show differences with sample-to-sample variations. Individual surface water samples may indeed deviate from the model (composite) sample in terms of constituents such as natural dissolved organic matter—while inorganic salts would be mostly removed during the on-line desalting process. Matrix effects for the tested surface water samples are provided in SI (S4 Table). The overall range of matrix effects when considering the different analyte/sample combinations was -22% to +17%, equating to sample-specific accuracies between 78% and 117%. This may be considered an acceptable range (accuracy within 70–120%) according to established guidelines [35,36].

### 3.6 Analytical method performance

The analytical method was validated following guidelines for single laboratory validation [36,37,63]. The obtained results are compiled in Table 2.

**Table 2:** Analytical method performance. Summary of the analytical performance in surface water matrix, including method limits of detection (LOD, ng L^-1^) and limits of quantification (LOQ, ng L^-1^), linearity range (ng L^-1^) and associated determination coefficients (R^2^), and accuracy (%) and precision (RSD%) at the two tested spike levels (QC_1_: 75 ng L^-1^; QC_2_: 750 ng L^-1^).

	AEG	BAMA	DAB	BMAA
LOD (ng L^-1^)	2	5	3	5
LOQ (ng L^-1^)	5	10	5	10
Linear range (ng L^-1^)	LOQ-1000	LOQ-1000	LOQ-1000	LOQ-1000
R^2^	0.9982	0.9963	0.9979	0.9973
Accuracy (%) at QC_1_	90 ± 5	95 ± 6	76 ± 2	91 ± 4
Accuracy (%) at QC_2_	92 ± 3	99 ± 5	101 ± 2	100 ± 3
Intraday precision (RSD%, n = 5) at QC_1_	5.9	6.7	2.6	4.3
Intraday precision (RSD%, n = 5) at QC_2_	3.5	4.8	2.1	2.7
Interday precision (RSD%, n = 15) at QC_1_	13.0	10.8	8.1	11.4
Interday precision (RSD%, n = 15) at QC_2_	5.8	5.0	4.9	4.9

Method limits of detection (LOD) and limits of quantification (LOQ) were assessed as per the EURACHEM [38] method (Section 2.5). Compound-specific LODs were in the range of 2–5 ng L^-1^, while LOQs were set at 5 ng L^-1^ for AEG and DAB, and 10 ng L^-1^ for BAMA and BMAA (Table 2). Few methods have been reported for the analysis of BMAA and its isomers in surface water apart from cyanobacterial blooms, which makes it difficult to compare our sensitivity performance with the literature since LODs/LOQs are often expressed on a μg g^-1^ dry weight basis and the sampled volumes are not always documented as noted in recent reviews [29]. The LODs reported here are in the same order of magnitude as a workflow involving off-line solid-phase extraction of lake water and dansyl chloride derivatization followed by LC-MS/MS [61], although the latter method derived a much higher sample intake (100 mL) compared to the present study (5 mL).

Matrix-matched internal calibration curves were produced with suitable linearity over the tested range of 5–1000 or 10–1000 ng L^-1^ (Table 2). The R^2^ were in the range of 0.9963–0.9982 and bias between expected and calculated-back concentration was typically within ±10% except for the low-end calibration curve level close to the LOQ (bias within ±25%). The observed performance is compliant with acceptance criteria (e.g., R^2^ ≥0.99).

According to performance acceptability criteria, accuracy should be within 70–120% and associated repeatability RSD <20% [35]. In the present study, accuracy was in the range of 76–95% and 92–101% at the two tested fortification levels (Table 2). Intraday precision (% RSD) was in the range of 2.6–6.7% and 2.1–4.8% in surface water samples spiked at 75 ng L^-1^ and 750 ng L^-1^, respectively. Interday precision remained in the overall range of 4.9–13%. The compliance to accuracy acceptability criterion was also verified along the LC-MS sequence, through regular analysis of CVC standards. We also observed low retention time variability across CVC standards (<0.2%) and satisfactory mass accuracy, as shown in SI (S5 Table).

### 3.7 Screening of AEG, BAMA, DAB, and BMAA in river and lake surface waters

The validated analytical method was applied to surface water samples, including sites with HAB and non-HAB contexts. When considering the overall data set (n = 82), DAB was the most recurrent compound (detection frequency of 73%), while AEG was also frequently reported (62%). BMAA was only reported in 1/82 samples and BAMA in 2/82 samples. Overall concentration ranges were as follows for the targeted compounds: AEG (<2–4,900 ng L^-1^), BAMA (<5–130 ng L^-1^), DAB (<3–1,900 ng L^-1^), and BMAA (<5–110 ng L^-1^). As one of the first reports on dissolved concentrations of BMAA and its isomers in surface water, a direct comparison of concentrations from the present study (ng L^-1^) with literature data was not feasible. The widespread occurrence of DAB is, however, in agreement with its systematic or near-systematic detections in cyanobacterial samples from Australia [11] and Canada [14], with concentrations ranging from μg g^-1^ to mg g^-1^ levels.

The highest concentrations observed in the present survey were from impacted lake samples (Table 3). For instance, surface water samples with DAB concentrations higher than 100 ng L^-1^ corresponded to the following locations: Buffalo Pound Lake (SK, scum, DAB = 100 ng L^-1^), Missisquoi Bay (QC, blooms, DAB = 110–450 ng L^-1^), Lac Memphrémagog (QC, DAB = 220 ng L^-1^), and Petit lac Saint-François (QC, bloom, DAB = 1,900 ng L^-1^). AEG displayed variable levels across samples, with a maximum of 4,900 ng L^-1^ measured for an HAB-impacted sample from Petit lac Saint-François (Table 3). BAMA was reported at a concentration of 40 ng L^-1^ in a crowd-sourcing sample from Lac Memphrémagog, for which elevated concentrations of cyanopeptides (microcystins, anabaenopeptins) were also measured (unpublished data). BMAA was only reported in one HAB-impacted sample from Quebec province (Missisquoi Bay, bloom, 110 ng L^-1^).

**Table 3:** Application to field samples—Lakes. Concentration ranges (ng L^-1^) of AEG, BAMA, DAB, and BMAA in selected lake samples (n = 61) with HAB monitoring activities.

			Concentration range (ng L^-1^)			
Site	Sampling years	Samples (n)	AEG	BAMA	DAB	BMAA
Buffalo Pound Lake	2017–2018	10	ND–29	ND	18–100	ND
Heart Lake	2017	1	ND	ND	ND	ND
Lake 227	2018	2	ND–2.1	ND	12–13	ND
Lake Bass	2017	1	8.4	ND	ND	ND
Lake Conestogo (site #1)	2018	2	2–3	ND	15–20	ND
Lake Conestogo (site #2)	2018	5	ND–3	ND	13–33	ND
Lake Eversley	2017	1	ND	ND	33	ND
Lake Fanning	2017	1	21	ND	25	ND
Lac McKay	2018	1	ND	ND	15	ND
Lac Memphrémagog	2018	1	8.9	41	220	ND
Lac Morin	2017	1	ND	ND	ND	ND
Lake Mulgrave	2017	1	12	ND	24	ND
Lake Napadogan	2017	1	ND	ND	ND	ND
Lake Nowlans	2017	1	8.5	ND	26	ND
Lake Ogden	2017	1	19	ND	24	ND
Lake Provost	2017	1	ND	ND	ND	ND
Lac Roxton	2018	1	2.5	ND	14	ND
Lac Saint-Charles	2017–2018	6	ND–19	ND	ND–26	ND
Lac Saint-Paul	2017	1	32	ND	63	ND
Lac Waterloo	2018	1	ND	ND	14	ND
Missisquoi Bay (site #2)	2016–2018	6	ND–14	ND–130	15–450	ND–110
Petit lac Saint-François	2017–2018	11	ND–4900	ND	ND–1900	ND
Philipsburg Littoral (Missisquoi Bay)	2016	1	2.5	ND	14	ND
Pike River	2017–2018	2	ND–72	ND	21–25	ND
Réservoir Choinière	2018	1	6.7	ND	18	ND
Overall range	2016–2018	61	<2–4900	<5–130	<3–1900	<5–110

Surface waters from non-HAB environments (n = 21) displayed consistently low occurrence and levels of the 4 targeted compounds (Table 4). BAMA and BMAA were not found in any of these riverine samples. In samples collected within the St. Lawrence River (n = 6), monoresidual detections were reported for 2 samples, while none of the targeted analytes were reported in the other 4 samples. The highest concentrations of AEG and DAB in tributary rivers (Table 4) were 25 and 16 ng L^-1^, respectively, more than 2 orders of magnitude lower than maximum observed concentrations from some HAB-impacted lakes (Table 3).

**Table 4:** Application to field samples—Rivers. Concentrations (ng L^-1^) of AEG, BAMA, DAB, and BMAA in background surface water samples (n = 21) collected in the St. Lawrence River watershed (QC, Canada).

	Concentration (ng L^-1^)			
Sampling site	AEG	BAMA	DAB	BMAA
St. Lawrence River #1	2.2	ND	ND	ND
St. Lawrence River #2	ND	ND	ND	ND
St. Lawrence River #3	ND	ND	ND	ND
St. Lawrence River #4	ND	ND	ND	ND
St. Lawrence River (Lac Saint-Pierre #1)	ND	ND	13	ND
St. Lawrence River (Lac Saint-Pierre #2)	ND	ND	ND	ND
Rivière à la Tortue	2.3	ND	14	ND
Rivière L’Assomption	5.4	ND	ND	ND
Rivière Bécancour	ND	ND	ND	ND
Rivière des Prairies	6.4	ND	ND	ND
Rivière du Loup	2.1	ND	13	ND
Rivière Jacques-Cartier	ND	ND	16	ND
Rivière Mascouche	ND	ND	13	ND
Rivière Maskinongé	ND	ND	ND	ND
Rivière des Mille Iles	8.1	ND	ND	ND
Rivière Nicolet	2.6	ND	ND	ND
Ottawa River	5.6	ND	ND	ND
Rivière Richelieu	3.6	ND	ND	ND
Rivière Saint-François	3.7	ND	ND	ND
Rivière Yamachiche	25	ND	ND	ND
Rivière Yamaska	4.5	ND	13	ND
Overall concentration range	<2–25	<5	<3–16	<5

## Conclusions

The developed analytical approach allows the screening of BMAA and its isomers at ultra-trace levels in surface water samples. The method is based on a small sample intake (5 mL), relatively short FMOC derivatization reaction (1h), and automated extraction coupled on-line to UHPLC-HRMS allowing to expedite the pre-concentration and instrumental detection process (10 min per sample). The on-line desalting step was the driving factor for suitable method robustness, in view of the high salt content of derivatized samples. The FMOC-grafted amino acids were successfully separated using reverse-phase liquid chromatography (C18) in combination with ammonium acetate mobile phases. Using this workflow, method limits of detection in the low part-per-trillion range were obtained (LOD: 2–5 ng L^-1^). Method validity was ascertained in spiked surface water samples, considering linearity, accuracy, and precision endpoints; the performance was compliant with internationally accepted criteria. We demonstrated the suitability of a matrix-matched calibration approach with internal standardization, with low sample-to-sample matrix effects.

After the initial method optimization and validation, the method has been applied to long sample sequences with suitable robustness and low back-pressure increase over consecutive injections. The profiles of AEG, BAMA, DAB, and BMAA were documented at large spatial scale for the first time in surface water samples. The Canadian survey covered shallow eutrophic lakes that are known to be prone to HAB events, but also river water samples from non-HAB contexts. Our results suggest a potential widespread occurrence of AEG and DAB, especially in bloom-impacted samples with concentrations surpassing μg L^-1^ levels. BMAA was only detected in 1 out of 82 monitored samples (110 ng L^-1^), also related to a cyanobacterial bloom. This suggests that BMAA can occur in such temperate cyanobacterial blooms but does not seem to be prevalent, while AEG and DAB are much more frequent. Future application of this method to a temporal follow-up of selected lakes, in conjunction with measurements of other cyanobacterial toxins and environmental parameters, could provide useful data toward the early prediction of toxic cyanobacterial blooms.

## Supporting information

S1 Table Field-collected surface water samples from HAB-impacted environments. (PDF)

S2 Table Field-collected surface water samples from background environments in the St. Lawrence watershed (QC, Canada). (PDF)

S3 Table Summary of UHPLC-HRMS instrumental method parameters. (PDF)

S4 Table Assessment of relative matrix effects (%) on surface waters from different locations. The relative matrix effects were derived from the standard additions slope to the particular sample, compared to the reference used for quantification (i.e., matrix-matched calibration curve).
(PDF)

S5 Table Retention time (average ± SD) and exact mass accuracy (Δppm) of the targeted analytes, evaluated on continued calibration verification (CCV) standards. Quality control spikes were run after every 15 injections in the LC-MS sequence. The relative standard deviation (RSD) of retention time was <0.2%, which is within the tolerance threshold of ±2.5% set by the European Commission (2002/657/EC). The Δppm tolerance applied was ± 5 ppm.
(PDF)

S1 Fig Schematic view of the sample preparation and analysis workflow. (PDF)

S2 Fig Influence of the addition of organic solvent prior FMOC derivatization reaction on the FMOC-BMAA signal. A surface water matrix was spiked at 1000 ng L^-1^ with BMAA and submitted to the different conditions. Absolute areas were normalized (%) to the maximum observed among the tested conditions. The final organic solvent percentage was adjusted to 15% in all samples prior LC-MS analysis. Error bars represent standard deviations (n = 3).
(PDF)

S3 Fig Influence of post-reactions conditions (including storage temperature for sample cool down and organic solvent addition) on the FMOC-BMAA signal. A surface water matrix was spiked at 1000 ng L^-1^ with BMAA and submitted to the different conditions. Absolute areas were normalized (%) to the maximum observed among the tested conditions. Error bars represent standard deviations (n = 3).
(PDF)

S4 Fig Illustration of the possible degradation of EDTA to form AEG. This could explain the blank backgrounds observed when using EDTA. In the present study, the use of EDTA was therefore avoided and we used citrate instead for metallic ions complexation.
(PDF)

S5 Fig Optimization of derivatization reaction: Influence of citrate buffer concentration on the FMOC-BMAA signal. A surface water matrix was spiked at 1000 ng L^-1^ with BMAA and submitted to the different conditions. Absolute areas were normalized (%) to the maximum observed among the tested conditions. Error bars represent standard deviations (n = 3).
(PDF)

S6 Fig Optimization of derivatization reaction: Influence of borate buffer concentration on the FMOC-BMAA signal. A surface water matrix was spiked at 1000 ng L^-1^ with BMAA and submitted to the different conditions. Absolute areas were normalized (%) to the maximum observed among the tested conditions. Error bars represent standard deviations (n = 3).
(PDF)

S7 Fig Optimization of derivatization reaction: Influence of FMOC-Cl concentration on the FMOC-BMAA signal. A surface water matrix was spiked at 1000 ng L^-1^ with BMAA and submitted to the different conditions. Absolute areas were normalized (%) to the maximum observed among the tested conditions. Error bars represent standard deviations (n = 3).
(PDF)

S8 Fig Selection of the on-line SPE column, illustrated for FMOC-BMAA. The two tested columns were as follows: HyperSep (HyperSep Retain PEP column, hydrophilic lipophilic balance; 20 mm x 2.1 mm; 40–60 μm particle size) and C18 (Hypersil Gold aQ C18; 20 mm x 2.1 mm; 12 μm particle size). Absolute areas were normalized (%) to the maximum observed among the tested conditions. Error bars represent standard deviations (n = 3).
(PDF)

S9 Fig Influence of the wash volume applied (desalting step) on analyte response. The wash volume was varied between 0.5 and 2.5 mL for matrix and salt removal of the derivatization samples. Absolute areas were normalized (%) to the maximum observed among the tested conditions. Error bars represent standard deviations (n = 3).
(PDF)

S10 Fig Influence of the on-line SPE flowrate on analyte signal. The flowrate was varied between 1,000 and 2,500 μL min^-1^. Absolute areas were normalized (%) to the maximum observed among the tested conditions. Error bars represent standard deviations (n = 3).
(PDF)

S11 Fig Influence of LC-MS sample injection loop nature (Peek *vs*. Stainless steel). Absolute areas were normalized (%) to the maximum observed among the tested conditions. Error bars represent standard deviations (n = 3).
(PDF)

S12 Fig Stability of FMOC-BMAA absolute area tested over 12.5h post-preparation. All samples were prepared and positioned on the LC-MS plate at T_0_, and analyzed after applying different wait times (0, 2, 4.5, 7, 8.5, 11, and 12.5h).
(PDF)
