# Supplementary material for: Analysis of the neurotoxin β-N-methylamino-L-alanine (BMAA) and isomers in surface water by FMOC derivatization liquid chromatography high resolution mass spectrometry
Source: PLoS One. 2019 Aug 6;14(8):e0220698. doi: 10.1371/journal.pone.0220698 (PMC6684067; doi:10.1371/journal.pone.0220698)
Supplement: S1 Table — (PDF) [file pone.0220698.s001.pdf]

S1 Table. Field-collected surface water samples from HAB-impacted environments.

Sample location	Province	Project	Sampling year	Sampling date or period
Buffalo Pound Lake	Saskatchewan	ATRAPP	2017	05/09/2017
Buffalo Pound Lake	Saskatchewan	ATRAPP	2017	05/10/2017
Buffalo Pound Lake	Saskatchewan	ATRAPP	2017	28/08/2017
Buffalo Pound Lake	Saskatchewan	ATRAPP	2017	25/08/2017
Buffalo Pound Lake	Saskatchewan	ATRAPP	2017	05/09/2017
Buffalo Pound Lake	Saskatchewan	ATRAPP	2018	12/07/2018
Buffalo Pound Lake	Saskatchewan	ATRAPP	2018	25/06/2018
Buffalo Pound Lake (scum)	Saskatchewan	ATRAPP	2018	19/06/2018
Buffalo Pound Lake (scum)	Saskatchewan	ATRAPP	2018	19/06/2018
Buffalo Pound Lake (scum)	Saskatchewan	ATRAPP	2018	21/06/2018
Heart Lake	Ontario	ATRAPP	2017	05/08/2017
Lake 227	Ontario	ATRAPP	2018	10/07/2018
Lake 227	Ontario	ATRAPP	2018	11/06/2018
Lake Bass	Ontario	ATRAPP	2017	26/08/2017
Lake Conestogo (central arm)	Ontario	ATRAPP	2018	04/09/2018
Lake Conestogo (central arm)	Ontario	ATRAPP	2018	27/07/2018
Lake Conestogo (east arm)	Ontario	ATRAPP	2018	07/09/2018
Lake Conestogo (east arm)	Ontario	ATRAPP	2018	17/09/2018
Lake Conestogo (east arm)	Ontario	ATRAPP	2018	25/06/2018
Lake Conestogo (east arm)	Ontario	ATRAPP	2018	03/07/2018
Lake Conestogo (east arm)	Ontario	ATRAPP	2018	26/07/2018
Lake Eversley	Ontario	ATRAPP	2017	03/08/2017
Lake Fanning	Nova Scotia	ATRAPP	2017	15/08/2017
Lac McKay	Quebec	Adopt a Lake	2018	22/08/2018
Lac Memphrémagog	Quebec	Adopt a Lake	2018	18/07/2018
Lac Morin	Quebec	ATRAPP	2017	24/07/2017
Lake Mulgrave	Nova Scotia	ATRAPP	2017	09/08/2017
Lake Napadogan	New Brunswick	ATRAPP	2017	02/08/2017
Lake Nowlans	Nova Scotia	ATRAPP	2017	29/08/2017
Lake Ogden	Nova Scotia	ATRAPP	2017	11/07/2017
Lake Provost	Nova Scotia	ATRAPP	2017	30/08/2017
Lac Roxton	Quebec	ATRAPP	2018	14/07/2018
Lac Saint-Charles	Quebec	ATRAPP	2018	11/06/2018
Lac Saint-Charles	Quebec	ATRAPP	2017	01/08/2017
Lac Saint-Charles	Quebec	ATRAPP	2017	13/09/2017
Lac Saint-Charles	Quebec	ATRAPP	2018	03/10/2018
Lac Saint-Charles	Quebec	ATRAPP	2018	04/09/2018
Lac Saint-Charles	Quebec	ATRAPP	2017	29/08/2017
Lac Saint-Paul	Quebec	ATRAPP	2017	20/08/2017
Lac Waterloo	Quebec	ATRAPP	2018	28/07/2018
Missisquoi Bay (site #2)	Quebec	ATRAPP	2016	October 2016
Missisquoi Bay (site #2)	Quebec	ATRAPP	2017	Summer 2017
Missisquoi Bay (site #2)	Quebec	ATRAPP	2018	20/08/2018
Missisquoi Bay (site #2)	Quebec	ATRAPP	2017	30/08/2017
Missisquoi Bay (site #2)	Quebec	ATRAPP	2017	2017-0720
Missisquoi Bay (site #2)	Quebec	ATRAPP	2017	07/08/2017
Petit lac Saint-François	Quebec	ATRAPP	2018	07/08/2018
Petit lac Saint-François	Quebec	ATRAPP	2018	29/05/2018
Petit lac Saint-François	Quebec	ATRAPP	2018	23/10/2018
Petit lac Saint-François	Quebec	ATRAPP	2018	04/09/2018
Petit lac Saint-François	Quebec	ATRAPP	2018	11/09/2018
Petit lac Saint-François	Quebec	ATRAPP	2018	20/09/2018
Petit lac Saint-François	Quebec	ATRAPP	2017	17/10/2017
Petit lac Saint-François	Quebec	ATRAPP	2017	31/08/2017
Petit lac Saint-François	Quebec	ATRAPP	2017	12/09/2017
Petit lac Saint-François	Quebec	ATRAPP	2018	06/06/2018
Petit lac Saint-François	Quebec	ATRAPP	2018	07/07/2018
Philipsburg Littoral (Missisquoi Bay)	Quebec	ATRAPP	2016	05/10/2016
Pike River (Bedford)	Quebec	ATRAPP	2018	31/07/2018
Pike River (Bedford)	Quebec	ATRAPP	2017	27/07/2017
Réservoir Choinière	Quebec	Adopt a Lake	2018	28/07/2018
