# Supplementary material for: Analysis of the neurotoxin β-N-methylamino-L-alanine (BMAA) and isomers in surface water by FMOC derivatization liquid chromatography high resolution mass spectrometry
Source: PLoS One. 2019 Aug 6;14(8):e0220698. doi: 10.1371/journal.pone.0220698 (PMC6684067; doi:10.1371/journal.pone.0220698)
Supplement: S2 Table — (PDF) [file pone.0220698.s002.pdf]

S2 Table. Field-collected surface water samples from background environments in the St. Lawrence watershed (QC, Canada).

Sample Code	Site location	Sampling period
St. Lawrence River #1	Lac Saint François, transect (4B)	July 2018 (*)
St. Lawrence River #2	Lac Saint Louis, transect (1B)	July 2018 (*)
St. Lawrence River #3	Iles de Boucherville, transect (1B)	July 2018 (*)
St. Lawrence River #4	Contrecœur, transect (3B)	July 2018 (*)
Lac Saint-Pierre #1	Lac Saint-Pierre, LSPM transect (3B)	July 2018 (*)
Lac Saint-Pierre #2	Lac Saint-Pierre, LSPA transect (3B)	July 2018 (*)
Rivière à la Tortue	Rivière à la Tortue (Parc à la Tortue, Delson)	October 2018
Rivière L'Assomption	Rivière L'Assomption (mouth)	September 2018
Rivière Bécancour	Rivière Bécancour (mouth)	September 2018
Rivière des Mille Iles	Rivière des Mille Iles (Bois des Filion)	October 2018
Ottawa River	Rivière des Outaouais (30 km upstream from the mouth)	September 2018
Rivière des Prairies	Rivière des Prairies (Parc Maurice Richard)	October 2018
Rivière du Loup	Rivière Du Loup (mouth)	September 2018
Rivière Jacques-Cartier	Rivière Jacques-Cartier (Donnacona)	October 2018
Rivière Mascouche	Rivière Mascouche (Terrebonne; mouth)	October 2018
Rivière Maskinongé	Rivière Maskinongé (mouth)	September 2018
Rivière Nicolet	Rivière Nicolet (mouth)	September 2018
Rivière Richelieu	Rivière Richelieu (Sorel-Tracy; mouth)	September 2018
Rivière Saint-François	Rivière Saint-François (mouth)	September 2018
Rivière Yamachiche	Rivière Yamachiche (mouth)	September 2018
Rivière Yamaska	Rivière Yamaska (mouth)	September 2018

(*) Samples collected with the Lampsilis (UQTR) research vessel.
