# Supplementary material for: Analysis of the neurotoxin β-N-methylamino-L-alanine (BMAA) and isomers in surface water by FMOC derivatization liquid chromatography high resolution mass spectrometry
Source: PLoS One. 2019 Aug 6;14(8):e0220698. doi: 10.1371/journal.pone.0220698 (PMC6684067; doi:10.1371/journal.pone.0220698)
Supplement: S3 Table — (PDF) [file pone.0220698.s003.pdf]

S3 Table. Summary of UHPLC-HRMS instrumental method parameters.

**Instrument**	Thermo Q-Exactive Orbitrap mass spectrometer<br>Dionex Ultimate 3000 UHPLC chain
**Ionization**	Electrospray ionization source (negative mode)
**Acquisition mode**	Full Scan MS (200-600 m/z)
**Analytical column**	Thermo Hypersil Gold C18 column (100 × 2.1 mm; 1.9 µm)
**Column Temperature**	35°C
**Analytical Mobile Phases**	A: 2.5 mM ammonium acetate in HPLC-water<br>B: acetonitrile<br>Flow rate (mL/min) 0.45
**Gradient Profile (analytical column)**	Time (min) % B<br>0.0 25<br>2.0 25<br>4.0 40<br>8.0 50<br>8.4 100<br>9.4 100<br>9.5 25<br>10 25
**Injection Volume**	2000 µL (on-line SPE)
**On-line SPE column**	Thermo HyperSep Retain PEP column (20 mm × 2.1 mm, 40–60 µm)
**On-line SPE Mobile Phases**	A: HPLC-water<br>B: acetonitrile<br>Flow rate (mL/min) 2
**Gradient Profile (on-line SPE)**	Time (min) % B<br>0.0 0<br>2.0 0<br>2.1 100<br>6.9 100<br>7.0 0<br>10 0
**Source/gas**	Sheath gas flow rate 55<br>Aux gas flow rate 10<br>Sweep gas flow rate 0<br>Spray voltage (|kV|) 3.5<br>Capillary temperature (°C) 320<br>Vaporizer temperature (°C) 350<br>S-lens RF level 70
**Orbitrap parameters**	Resolution 70,000<br>AGC target 3e6<br>Maximum Inject Time (ms) 100
