# Supplementary material for: Analysis of the neurotoxin β-N-methylamino-L-alanine (BMAA) and isomers in surface water by FMOC derivatization liquid chromatography high resolution mass spectrometry
Source: PLoS One. 2019 Aug 6;14(8):e0220698. doi: 10.1371/journal.pone.0220698 (PMC6684067; doi:10.1371/journal.pone.0220698)
Supplement: S4 Table — The relative matrix effects were derived from the standard additions slope to the particular sample, compared to the reference used for quantification (i.e., matrix-matched calibration curve). (PDF) [file pone.0220698.s004.pdf]

S4 Table. Assessment of relative matrix effects (%) on surface waters from different locations. The relative matrix effects were derived from the standard additions slope to the particular sample, compared to the reference used for quantification (i.e., matrix-matched calibration curve).

	Relative matrix effect (%)		
	Lake St. Francis	Du Loup River	Richelieu River
AEG	2.1	1	11
BAMA	-3.7	-0.9	17
DAB	-17	-13	-14
BMAA	-7.0	-17	-22
