# Supplementary material for: Analysis of the neurotoxin β-N-methylamino-L-alanine (BMAA) and isomers in surface water by FMOC derivatization liquid chromatography high resolution mass spectrometry
Source: PLoS One. 2019 Aug 6;14(8):e0220698. doi: 10.1371/journal.pone.0220698 (PMC6684067; doi:10.1371/journal.pone.0220698)
Supplement: S5 Table — Quality control spikes were run after every 15 injections in the LC-MS sequence. The relative standard deviation (RSD) of retention time was <0.2%, which is within the tolerance threshold of ±2.5% set by the European Commission (2002/657/EC). The Δppm tolerance applied was ± 5 ppm. (PDF) [file pone.0220698.s005.pdf]

S5 Table. Retention time (average ± SD) and exact mass accuracy (∆ppm) of the targeted analytes, evaluated on continued calibration verification (CCV) standards. Quality control spikes were run after every 15 injections in the LC-MS sequence. The relative standard deviation (RSD) of retention time was <0.2%, which is within the tolerance threshold of ±2.5% set by the European Commission (2002/657/EC). The ∆ppm tolerance applied was ± 5 ppm.

	Retention time (min)	Average measured m/z	Theoretical m/z	∆ppm
AEG	6.29 ± 0.007	561.20417 ± 0.00007	561.20201	3.9
BAMA	6.45 ± 0.005	561.20419 ± 0.00009	561.20201	3.9
DAB	6.54 ± 0.007	561.20427 ± 0.00006	561.20201	4.0
BMAA	6.65 ± 0.012	561.20437 ± 0.00009	561.20201	4.2
