# Supplementary figures and images for: Analysis of the neurotoxin β-N-methylamino-L-alanine (BMAA) and isomers in surface water by FMOC derivatization liquid chromatography high resolution mass spectrometry
Source: PLoS One. 2019 Aug 6;14(8):e0220698. doi: 10.1371/journal.pone.0220698 (PMC6684067; doi:10.1371/journal.pone.0220698)

**S1 Fig. Schematic view of the sample preparation and analysis workflow.**

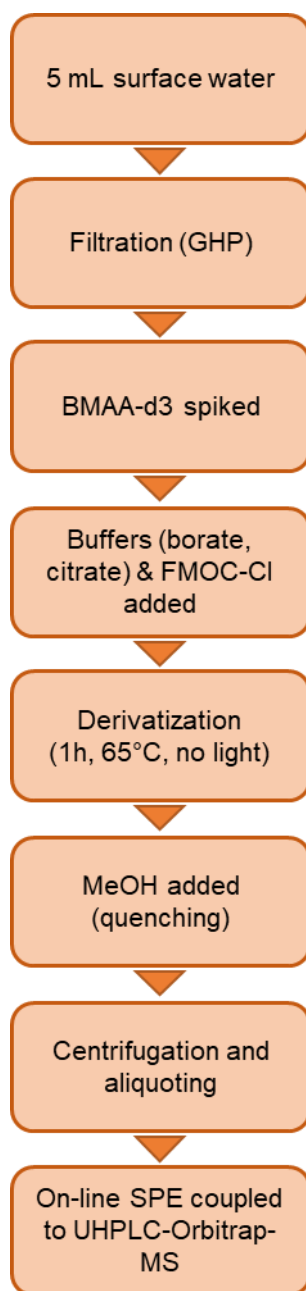

Supplement: S1 Fig — (PDF) [file pone.0220698.s006.pdf]
