# Supplementary material for: Analysis of the neurotoxin β-N-methylamino-L-alanine (BMAA) and isomers in surface water by FMOC derivatization liquid chromatography high resolution mass spectrometry
Source: PLoS One. 2019 Aug 6;14(8):e0220698. doi: 10.1371/journal.pone.0220698 (PMC6684067; doi:10.1371/journal.pone.0220698)

S3 Fig. Influence of post-reactions conditions (including storage temperature for sample cool down and organic solvent addition) on the FMOC-BMAA signal. A surface water matrix was spiked at 1000 ng L^{-1} with BMAA and submitted to the different conditions. Absolute areas were normalized (%) to the maximum observed among the tested conditions. Error bars represent standard deviations (n = 3).

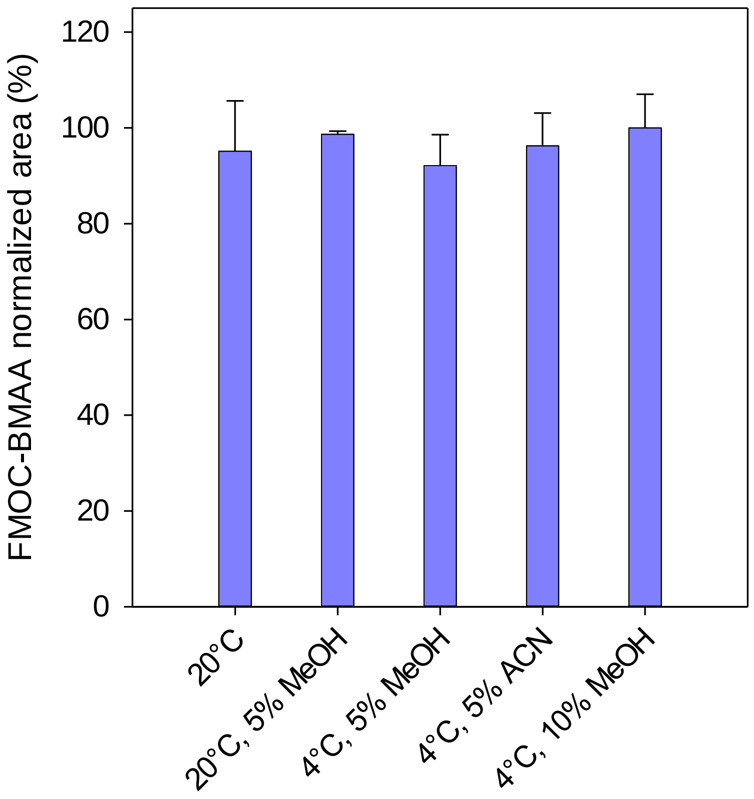

Supplement: S3 Fig — A surface water matrix was spiked at 1000 ng L-1 with BMAA and submitted to the different conditions. Absolute areas were normalized (%) to the maximum observed among the tested conditions. Error bars represent standard deviations (n = 3). (PDF) [file pone.0220698.s008.pdf]
