# Supplementary material for: Analysis of the neurotoxin β-N-methylamino-L-alanine (BMAA) and isomers in surface water by FMOC derivatization liquid chromatography high resolution mass spectrometry
Source: PLoS One. 2019 Aug 6;14(8):e0220698. doi: 10.1371/journal.pone.0220698 (PMC6684067; doi:10.1371/journal.pone.0220698)
Supplement: S4 Fig — This could explain the blank backgrounds observed when using EDTA. In the present study, the use of EDTA was therefore avoided and we used citrate instead for metallic ions complexation. (PDF) [file pone.0220698.s009.pdf]

S4 Fig. Illustration of the possible degradation[1,2] of EDTA to form AEG. This could explain the blank backgrounds observed when using EDTA. In the present study, the use of EDTA was therefore avoided and we used citrate instead for metallic ions complexation.

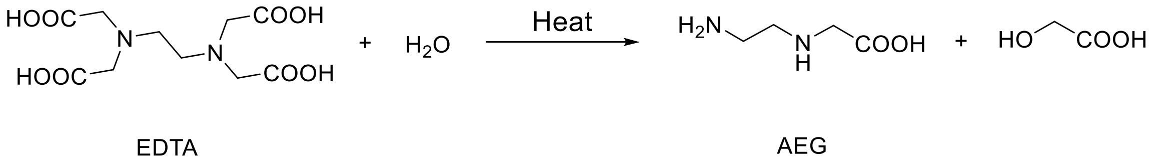

1. Motekaitis, R.J., Cox III, X.B., Taylor, P., Martell, A.E., Miles, B., Tvedt Jr, T.J., **1982**. Thermal degradation of EDTA chelates in aqueous solution. *Can. J. Chem.*, 60, 1207-1213.
2. Wang, J., Wang, X., Li, G., Guo, P., Luo, Z., **2010**. Degradation of EDTA in aqueous solution by using ozonolysis and ozonolysis combined with sonolysis. *J. Hazard. Mater.*, 176, 333-338.
