# Supplementary material for: Analysis of the neurotoxin β-N-methylamino-L-alanine (BMAA) and isomers in surface water by FMOC derivatization liquid chromatography high resolution mass spectrometry
Source: PLoS One. 2019 Aug 6;14(8):e0220698. doi: 10.1371/journal.pone.0220698 (PMC6684067; doi:10.1371/journal.pone.0220698)

S6 Fig. Optimization of derivatization reaction: influence of borate buffer concentration on the FMOC-BMAA signal. A surface water matrix was spiked at 1000 ng L^{-1} with BMAA and submitted to the different conditions. Absolute areas were normalized (%) to the maximum observed among the tested conditions. Error bars represent standard deviations (n = 3).

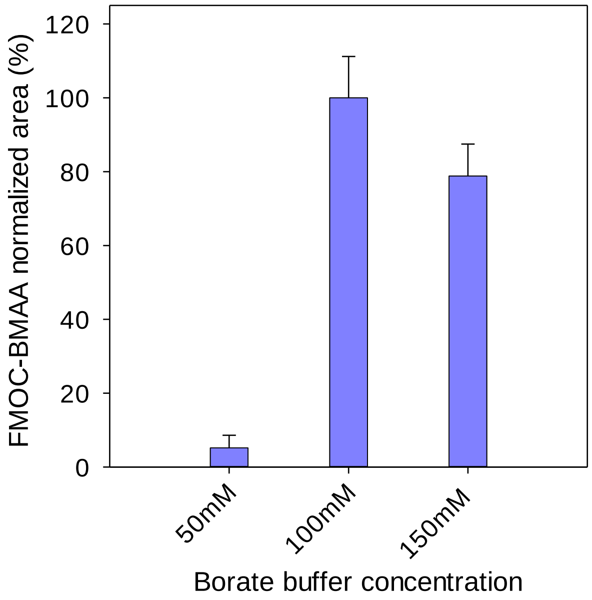

Supplement: S6 Fig — A surface water matrix was spiked at 1000 ng L-1 with BMAA and submitted to the different conditions. Absolute areas were normalized (%) to the maximum observed among the tested conditions. Error bars represent standard deviations (n = 3). (PDF) [file pone.0220698.s011.pdf]
