# Supplementary material for: Analysis of the neurotoxin β-N-methylamino-L-alanine (BMAA) and isomers in surface water by FMOC derivatization liquid chromatography high resolution mass spectrometry
Source: PLoS One. 2019 Aug 6;14(8):e0220698. doi: 10.1371/journal.pone.0220698 (PMC6684067; doi:10.1371/journal.pone.0220698)

S8 Fig. Selection of the on-line SPE column, illustrated for FMOC-BMAA. The two tested columns were as follows: HyperSep (HyperSep Retain PEP column, hydrophilic lipophilic balance; 20 mm x 2.1 mm; 40–60 µm particle size) and C18 (Hypersil Gold aQ C18; 20 mm x 2.1 mm; 12 µm particle size). Absolute areas were normalized (%) to the maximum observed among the tested conditions. Error bars represent standard deviations (n = 3).

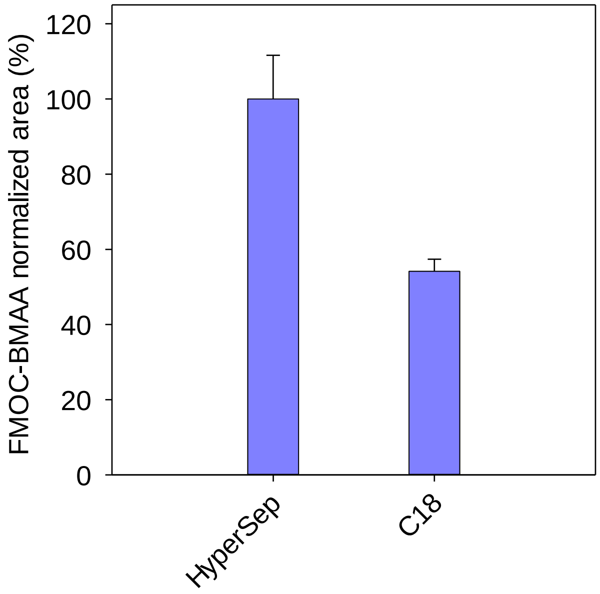

Supplement: S8 Fig — The two tested columns were as follows: HyperSep (HyperSep Retain PEP column, hydrophilic lipophilic balance; 20 mm x 2.1 mm; 40–60 μm particle size) and C18 (Hypersil Gold aQ C18; 20 mm x 2.1 mm; 12 μm particle size). Absolute areas were normalized (%) to the maximum observed among the tested conditions. Error bars represent standard deviations (n = 3). (PDF) [file pone.0220698.s013.pdf]
