# Supplementary material for: Analysis of the neurotoxin β-N-methylamino-L-alanine (BMAA) and isomers in surface water by FMOC derivatization liquid chromatography high resolution mass spectrometry
Source: PLoS One. 2019 Aug 6;14(8):e0220698. doi: 10.1371/journal.pone.0220698 (PMC6684067; doi:10.1371/journal.pone.0220698)

S9 Fig. Influence of the wash volume applied (desalting step) on analyte response. The wash volume was varied between 0.5 and 2.5 mL for matrix and salt removal of the derivatization samples. Absolute areas were normalized (%) to the maximum observed among the tested conditions. Error bars represent standard deviations (n = 3).

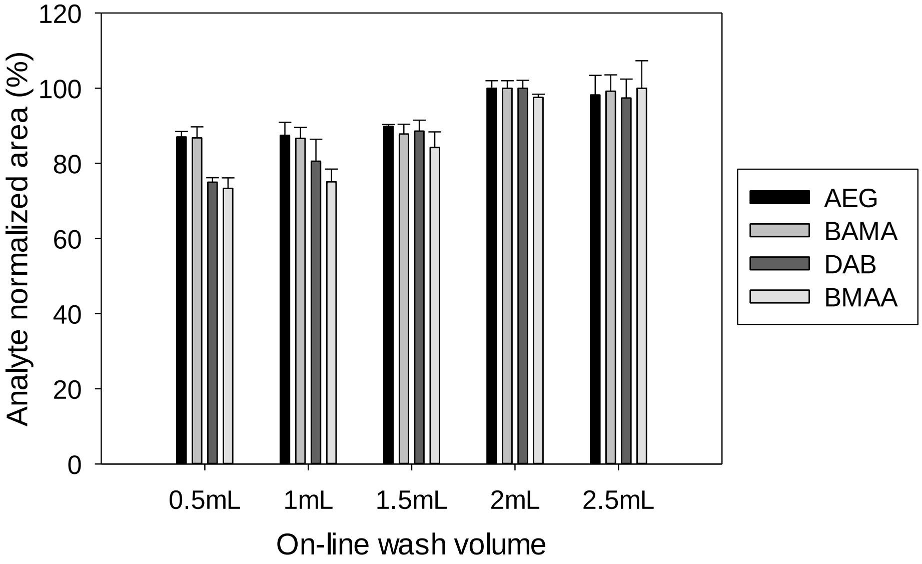

Supplement: S9 Fig — The wash volume was varied between 0.5 and 2.5 mL for matrix and salt removal of the derivatization samples. Absolute areas were normalized (%) to the maximum observed among the tested conditions. Error bars represent standard deviations (n = 3). (PDF) [file pone.0220698.s014.pdf]
