# Supplementary material for: Analysis of the neurotoxin β-N-methylamino-L-alanine (BMAA) and isomers in surface water by FMOC derivatization liquid chromatography high resolution mass spectrometry
Source: PLoS One. 2019 Aug 6;14(8):e0220698. doi: 10.1371/journal.pone.0220698 (PMC6684067; doi:10.1371/journal.pone.0220698)

S10 Fig. Influence of the on-line SPE flowrate on analyte signal. The flowrate was varied between 1,000 and 2,500 µL min^{-1}. Absolute areas were normalized (%) to the maximum observed among the tested conditions. Error bars represent standard deviations (n = 3).

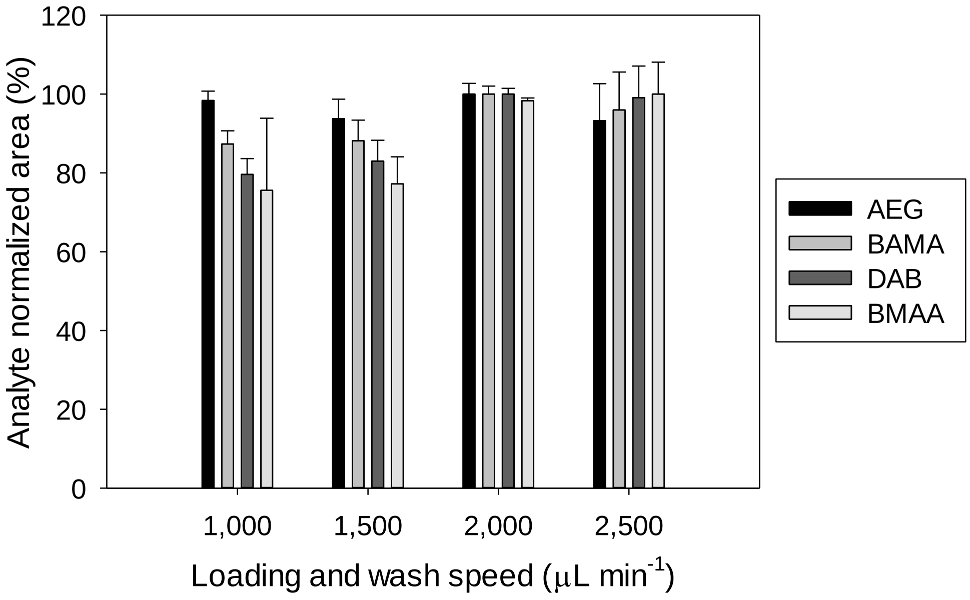

Supplement: S10 Fig — The flowrate was varied between 1,000 and 2,500 μL min-1. Absolute areas were normalized (%) to the maximum observed among the tested conditions. Error bars represent standard deviations (n = 3). (PDF) [file pone.0220698.s015.pdf]
