# Supplementary material for: Analysis of the neurotoxin β-N-methylamino-L-alanine (BMAA) and isomers in surface water by FMOC derivatization liquid chromatography high resolution mass spectrometry
Source: PLoS One. 2019 Aug 6;14(8):e0220698. doi: 10.1371/journal.pone.0220698 (PMC6684067; doi:10.1371/journal.pone.0220698)

S12 Fig. Stability of FMOC-BMAA absolute area tested over 12.5h post-preparation. All samples were prepared and positioned on the LC-MS plate at T_0, and analyzed after applying different wait times (0, 2, 4.5, 7, 8.5, 11, and 12.5h).

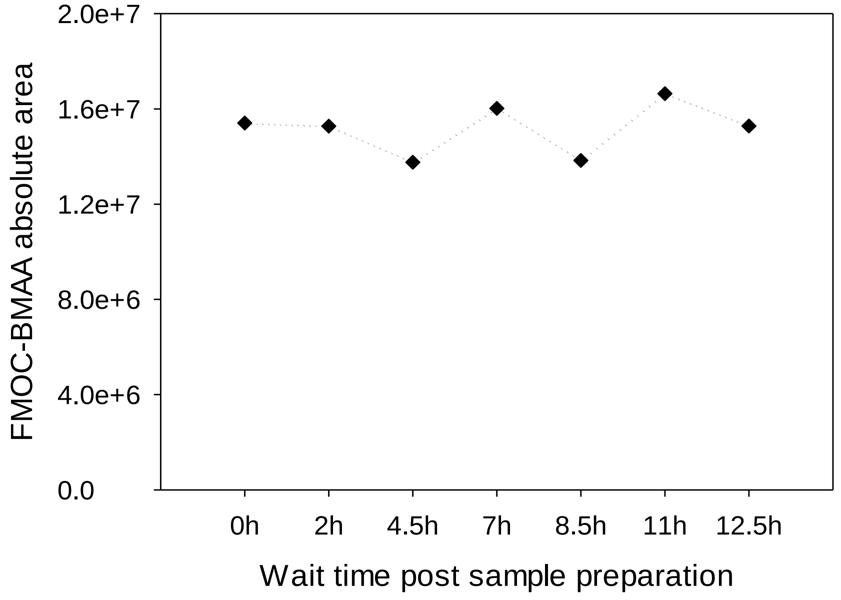

Supplement: S12 Fig — All samples were prepared and positioned on the LC-MS plate at T0, and analyzed after applying different wait times (0, 2, 4.5, 7, 8.5, 11, and 12.5h). (PDF) [file pone.0220698.s017.pdf]
